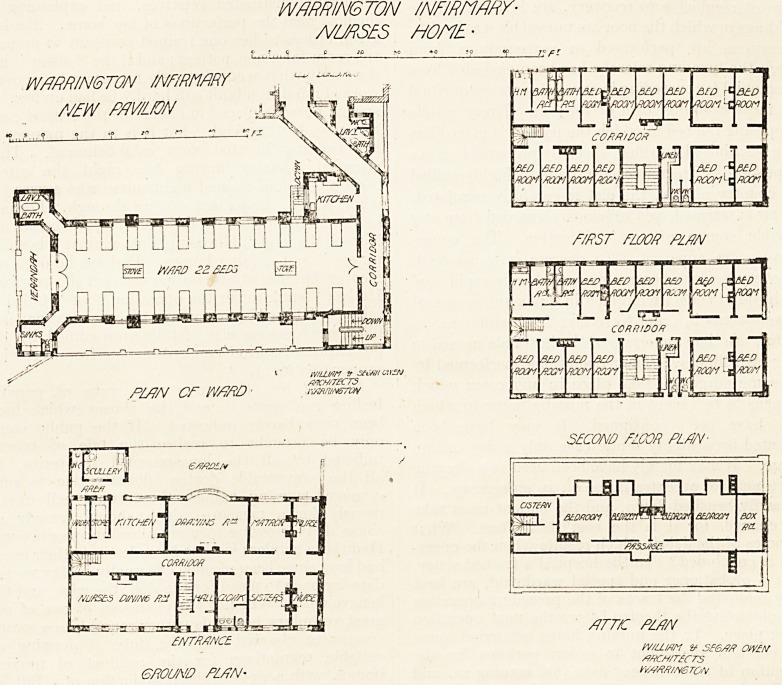# Warrington Infirmary

**Published:** 1907-11-16

**Authors:** 


### WARRINGTON INFIRMARY.

### NEW WARD PAVILION AND NURSES' HOME.

The additions to this institution which we illustrate to-day consist of a ward pavilion, giving an increase of 66 beds, and a detached nurses' home.

The ward pavilion is three floors in height and contains on each floor a ward for 22 beds, with ward kitchen, staircase, a bath-room and sanitary offices. The ward is connected with the main building by a corridor, and a sanitary annex has been added to the old wards on three floors. In the absence of a general plan showing the relation of the new wards to the old buildings and to the boundaries of the site it is somewhat difficult to criticise. The positions of the ward kitchen and of the staircase interfere seriously with the cross ventilation at the entrance end of the ward, and there is no reason apparent on the plan to prevent these adjuncts being to the north of the corridor, and so leaving the end of the ward free.

The sanitary offices appear unnecessarily cramped; one w.c. is not sufficient for 22 beds, and the bath-room is certainly not conveniently planned for manœuvring a helpless patient. The whole south end appears to have been copied from the Derby plan, with some essential features (*e.g.*, the small windows between the end beds and the doors to the sanitary annexes) omitted. The staircase is, we presume, for service only, its dimensions being too confined to admit of patients being carried up or down.

The wards are provided with two pairs of double ward-grates with descending flues, and also with hot-water pipes and radiators. Either the grates alone or the radiators alone are calculated to provide the requisite warmth. The floors are fireproof in construction, and are laid with pitch-pine boards, secret nailed and polished.

The nurses' home provides accommodation for the matron, two sisters, and 24 nurses. The building appears to be built with one end (no points of the compass are shown) on the boundary, from which it results that the corridor is lighted from one end only. The arrangement of the staircases is not happy. If two staircases were required they should have been placed at opposite ends of the building. They would then have become available as alternate modes of escape in case of fire. From the provision of a dining-room and kitchen it would seem that the home is complete in itself—a not very usual plan in homes of this size. Whether it is an economical plan to have a separate kitchen for the nurses or not is a point that cannot be discussed without more complete knowledge of the circumstances. Another point which is open to argument is the plan adopted here of putting the matron's rooms in the home. It is the view of many experienced matrons that the nurses when off duty should be in a measure free from the matron's surveillance; this can never be when the matron's rooms are in the nurses' home.

The architects for the two buildings described are Messrs. William and Segar Owen, of Warrington.

WARRINGTON INFIRMARY. NURSES HOME.

## Figures and Tables

**Figure f1:**